# Prevalence and risk factors for caesarean delivery following labor induction at a tertiary hospital in North Tanzania: a retrospective cohort study (2000–2015)

**DOI:** 10.1186/s12884-020-02861-8

**Published:** 2020-03-18

**Authors:** Clifford Silver Tarimo, Michael J. Mahande, Joseph Obure

**Affiliations:** 1 Department of Science and Laboratory Technology, Dar es Salaam Institute of Technology, P.O. Box 2958, Dar es Salaam, Tanzania; 2 Institute of Public Health, Kilimanjaro Christian Medical University College, P.O. Box 2240, Moshi, Tanzania; 3 Department of Obstetrics and Gynaecology, Kilimanjaro Christian Medical Centre, P.O. Box 3010, Moshi, Tanzania

**Keywords:** Labour induction, Caesarean section, Risk factors, North Tanzania

## Abstract

**Background:**

Labor induction is among the common and widely practiced obstetric interventions aiming at achieving vaginal delivery. However, cesarean section (CS) delivery incidences have been reported following its use. This study aimed at determining the prevalence and risk factors for caesarean delivery following labor induction among women who gave birth at a tertiary hospital in north-Tanzania.

**Methods:**

A hospital-based retrospective cohort study was designed using maternally-linked data from Kilimanjaro Christian Medical Centre (KCMC) birth registry among women who gave birth to singleton babies from the year 2000 to 2015. All induced deliveries done in this period were studied. Women with multiple pregnancy, missing information on delivery mode and those with history of CS delivery were excluded. Relative risk and 95% Confidence Interval for risk factors for CS delivery following labor induction were estimated using log-binomial regression models. Robust variance estimation was used to account for repeated deliveries from the same subject.

**Results:**

A total of 1088 deliveries were analysed. The prevalence of CS following labour induction was 26.75%. Independent risk factors for CS delivery were; primiparity (RR = 1.46; 95% CI: 1.18–1.81), high birthweight (RR =1.28; 95% CI: 1.02–1.61), post-term pregnancy (RR = 1.45; 95% CI: 1.09–1.93), and urban residence (RR =1.29; 95%CI: 1.05–1.58).

**Conclusion:**

In patients undergoing labor induction, primiparity, high birthweight, post dates and urban residence were found to associate with an elevated risk of caesarean delivery. Assessment of these factors prior to labor induction intervention is warranted to reduce adverse pregnancy outcomes associated with emergency caesarean delivery.

## Background

Efforts to attain maternal health-related Sustainable Development Goal (SDG) which aims at ensuring healthy lives and promote wellbeing for all at all ages, are still not well satisfactory in most of sub-Saharan countries including Tanzania [1, 2]. However, a number of obstetric interventions including labor induction (IOL) have been practiced to save lives of mothers and the unborn. Being one of life-serving interventions in obstetrics, IOL can; decrease frequency of still births, reduce risks of infection, and lower caesarean section (CS) rates without increasing adverse pregnancy outcomes [3, 4]. WHO recommends IOL procedure to be done only when it is more advantageous to terminate the pregnancy than to let it progress and it also recommends non-clinical interventions to reduce unnecessary CS delivery [5]. As the main goal of IOL is to help the mother to start labor and attain vaginal delivery, the intervention may fail to achieve this goal and hence necessitate CS intervention [6, 7]. CS is a medical procedure which involves delivery of a baby through an incision made in the mother’s abdomen and uterus [8, 9]. The frequency of CS has been steadily increasing globally in the past several decades with a rate of 32.8% [10]. Reasons that have been reported to contribute to this rise include; emergence of pregnancies with multiple gestations, rise of pregnancy complications, gestational obesity, previous CS, twin pregnancy, failure of progress in labor, breech presentation, maternal request and increase in rate of labor induction [10–12]. Just like the increase of CS deliveries, deliveries that include IOL have also been reported to increase worldwide such that, more than one in five pregnant women underwent IOL in the year 2009 [13]. Several studies have found the association between IOL and CS rates [14–16]. The contribution of IOL to CS rates is still unknown especially in most countries in Sub Saharan Africa including Tanzania. However, studies elsewhere have realized a 20% contribution of IOL to emergency CS [17]. Adverse effects of CS compared to vaginal delivery include; higher costs of surgery, slower recovery for the woman, increased risk of adverse events in subsequent pregnancies, increased rate of infections, injury to nearby organs, an increased need for blood transfusion and death [18–20].

## Methods

### Study setting

This study was carried out at the Kilimanjaro Christian Medical Centre (KCMC) located in Moshi urban district, in north Tanzania. KCMC is one of the four zonal referral hospitals that serves not only the residents of Kilimanjaro region but also Tanga, Manyara and Arusha regions. About 4000 deliveries are recorded annually at this facility. 20% of these admissions are referral cases while the remaining proportion comprises of self-referrals. Since the establishment of the birth registry at KCMC in 2000, the hospital has been recording information on pregnancy, delivery as well as information on the new-born in a separate electronic database. This information is obtained through personal interviews conducted by specially trained nurse-midwives either within 24 h after delivery in case of uncomplicated pregnancies or on the second or third day in case of CS delivery and other pregnancy complications. Major themes in the questionnaire include socio-demographic attributes of the child’s mother and father and various factors related to health status. The completed questionnaires are recorded and maintained in the computerized database.

### Study design

**Fig. 1:**
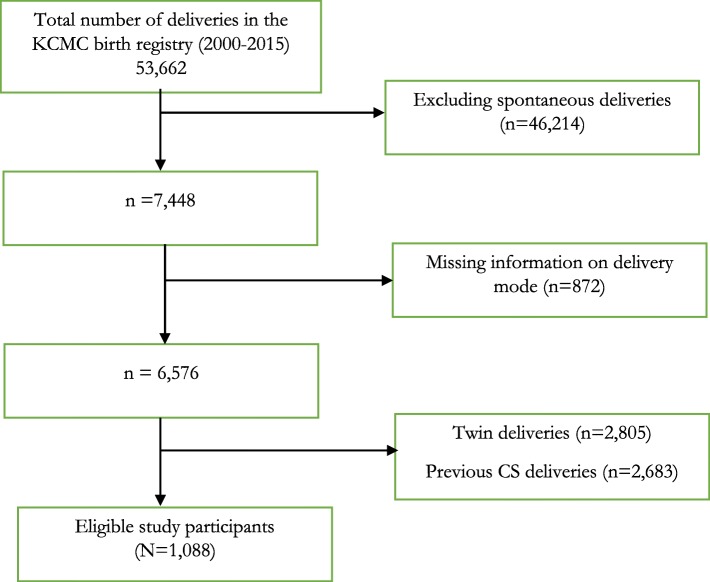
Schematic diagram for selected study participants

A retrospective cohort study was designed using maternally-linked data from KCMC-medical birth registry. We restricted our study to deliveries that were intervened by labor induction at KCMC hospital during the year 2000 to 2015. We excluded deliveries with multiple pregnancy (*n* = 2805) and those with a history of CS (*n* = 2683) so as to avoid overestimating the prevalence and risk factors for CS delivery following IOL intervention. Subjects with missing information on delivery mode achieved (*n* = 872) were also excluded since this attribute was used as the main study outcome of interest. We ended up with 1088 deliveries that complied to our eligibility criteria set in the current study (Fig. 1).

### Data source

In the year 2000, the medical birth registry at KCMC was launched in collaboration with the Medical Birth Registry of Norway and the University of Bergen. Records from the hospital registry database captures information regarding maternal socio-demographic characteristics, health status of the mother before and during pregnancy as well as information concerning delivery. Socio-demographic information that was extracted include information included age, occupation, education level, place of residence, marital status, tribe and religion. Clinical data on delivery explored include parity status, gestational age, mode of delivery, use of induction, indications for IOL, methods used for induction and body mass index (BMI). Well-trained midwife nurses conduct interviews on daily basis for every woman who gave birth at the facility by using a standardized questionnaire. Further description of the KCMC medical birth registry has been shown elsewhere [21].

### Statistical analysis

Data analysis was performed using STATA/IC (version 14, College Station, TX). Mean and standard deviation (SD) was used to describe continuous variables. Comparison of proportions was performed by Pearson chi-square (χ2) for categorical variables to determine associations between selected covariates and delivery mode. Multivariable log-binomial regression models were used to estimate Relative Risks (RRs) for CS following IOL with 95% confidence intervals (CIs). A *p*-value of less than 5% (2-tailed) was considered statistically significant for univariate and multivariable analyses of risk factors for CS delivery. We used delivery as the primary unit for our analysis and conducted a clustered analysis technique with robust estimation of variances to account for the correlation between repeated deliveries from the same woman.

## Results

A total of 1088 deliveries were analysed. Demographic characteristics of study participants are described in Table 1. The mean maternal age was 28 (SD = 6) years and majority (52%) of participants were aged between 20 and 30 years. More than half of women had either primary level education or not educated and more than 80% were married. Majority of the study subjects were of the indigenous of the study area, Chagga tribe (51.38%) and had normal body weight (34.2%). Table 1 has displayed the distribution of delivery mode achieved against maternal sociodemographic characteristics.

**Table 1:** Socio-demographic characteristics of study participants (*N* = 1088)

Characteristic	Total (N = 1088)	Cesarean delivery n (%)	Vaginal delivery n(%)	Chi-squared ***p***-value
**Maternal age (years)**				
< 20	85 (7.81)	23 (7.90)	62 (7.78)	0.345
20–30	567 (52.11)	164 (56.36)	403 (50.56)	
30–35	257 (23.62)	60 (20.62)	197 (24.72)	
> 35	179 (16.45)	44 (15.12)	135 (16.94)	
**Education status**				
None	28 (2.57)	8 (2.75)	20 (2.51)	0.909*
Primary	581 (53.40)	151 (51.89)	430 (53.95)	
Secondary	137 (12.59)	38 (13.06)	99 (12.42)	
Higher	339 (31.16)	94 (32.30)	245 (30.74)	
Missing	3 (0.28)	0 (0.00)	3 (0.38)	
**Religion**				
Catholic	379 (34.83)	112 (38.49)	267 (33.50)	0.167*
Protestant	437 (40.17)	121 (41.58)	316 (39.65)	
Muslim	263 (24.17)	57 (19.59)	206 (25.85)	
Others	9 (0.83)	1 (0.34)	8 (1.00)	
**Mother’s tribe**				
Chagga	559 (51.38)	165 (56.70)	394 (49.44)	0.068
Pare	148 (13.60)	31 (10.65)	117 (14.68)	
Others	381 (35.02)	95 (32.65)	286 (35.88)	
**Marital status**				
Married	916 (84.19)	243 (83.51)	673 (84.44)	0.708
Single	172 (15.81)	48 (16.49)	124 (15.56)	
**Residence**				
Rural	503 (46.23)	117 (40.21)	386 (48.43)	0.016
Urban	585 (53.77)	174 (59.79)	411 (51.57)	

*Fisher’s exact *p*-values

Obstetric characteristics of study participants are described in Table 2. More than half (53%) of all deliveries were from primiparous women. The use of oxytocin in inducing deliveries accounted for about 82% of all deliveries followed by prostaglandins (13%). Majority (58%) of the pregnant women were at term at the time of labor induction. Most (73.25%) of deliveries were attained vaginally. The most frequent indication for labor induction was post-date (88.7%), oxytocin being the most common (82.08%) method for induction used at this facility. Women with normal BMI had the highest proportion of CS delivery compared to other BMI categories. Increase in the infant birth weight was related to an increase in of CS delivery. 18% of deliveries involved big babies (> 3.5Kg) and 39% of these were delivered through CS. Primiparous women showed higher proportion of CS rate (32%) compared to multiparous women (21%).

**Table 2:** Distribution of clinical characteristics by delivery mode following labor induction

Characteristic	Total (***N*** = 1088)	Caesarean delivery n (%)	Vaginal deliver n (%)	Chi-square ***p***-value
**Gestational age**				
Term	632 (58.09)	198 (68.04)	434 (54.45)	< 0.001
Preterm	321 (29.50)	45 (15.46)	276 (34.63)	
Post term	63 (5.79)	31 (10.65)	32 (4.02)	
Missing	72 (6.62)	17 (5.84)	55 (6.90)	
**Indications for IOL**				
PROM	123 (11.31)	12 (4.12)	111 (13.93)	< 0.001
Post dates	965 (88.69)	279 (95.88)	686 (86.07)	
**Methods of Induction**				
Amniotomy	1 (0.09)	1 (0.34)	0 (0.00)	< 0.001*
Oxytocin	893 (82.08)	250 (85.91)	643 (80.68)	
Prostaglandins	142 (13.05)	21 (7.22)	121 (15.18)	
Missing	52 (4.78)	19 (6.53)	33 (4.14)	
**Parity**				
Primiparity	573 (52.67)	181 (62.20)	392 (49.18)	< 0.001
Multiparity	515 (47.33)	110 (37.80)	405 (50.82)	
**Body Mass Index**				
Underweight	167 (15.35)	39 (13.40)	128 (16.06)	0.226
Normal	360 (33.09)	108 (37.11)	252 (31.62)	
Overweight	199 (18.29)	52 (17.87)	147 (18.44)	
Obese	288 (26.47)	72 (24.74)	216 (27.10)	
Missing	74 (6.80)	20 (6.87)	54 (6.78)	
**Birthweights**				
Low	368 (33.82)	54 (18.56)	314 (39.40)	< 0.001
Normal	523 (48.07)	161 (55.33)	362 (45.42)	
High	197 (18.11)	76 (26.12)	121 (15.18)	

*PROM* Pre-labor rupture of membrane, *IOL* Induction of labor

*Fisher’s exact *p*-value

### Prevalence and trend of caesarean delivery following labor induction

**Fig. 2:**
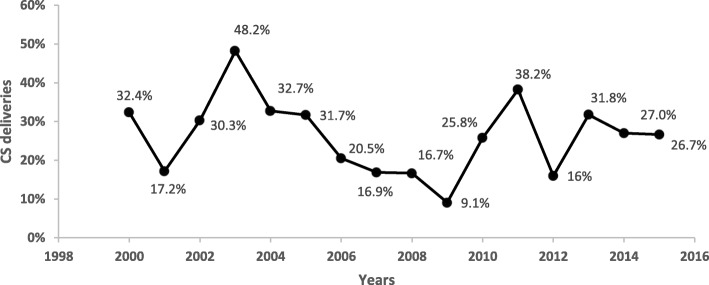
Trend of proportions of cesarean section delivery (CS) following labor induction during the year 2000 to 2015

Among 1088 induced deliveries analysed, 291 (26.75%) deliveries underwent CS. During the study period, nonuniform trend of CS delivery in induced delivery was appreciated. Most peaks were seen in the years 2003 (48.2%) and 2011 (38.2%) while in the year 2009, the study found the lowest prevalence of 9.1%.There was a variation in frequency of CS delivery by parity, gestational age, maternal BMI, and birth weight as displayed in Table 2. The trend of CS events is described in Fig. 2.

### Risk factors for CS delivery following labor induction

The crude and adjusted effects for predictors of CS delivery following labor induction have been displayed in Table 3**.** In the adjusted analyses, primiparous women were seen to have 40% increased risk of attaining CS delivery compared to multiparous women. We also found that, women who were post term (> 42 weeks gestation age) at the time of induction had more than 50% risk of experiencing CS intervention compared to those whose pregnancy were at term during labor induction. We did not find a significant association between maternal BMI and CS delivery. The study also found a statistically significant association between the birth weights and the risk of CS. We found that, the more the weight of the foetus the higher the likelihood of a mother to give birth by CS following IOL. High birth weight (≥3.5 kg) was associated with 24% increased risk of CS compared to normal birth weight (2.5–3.5 Kg). The adjusted analysis for association between mother’s residence and CS delivery shows that being in urban locality subjects a mother to 29% increased risks of CS intervention following IOL intervention compared to those living in rural areas (Table 3).

**Table 3:** Crude and adjusted effects on predictors of CS delivery following labor induction

Characteristic	Crude RR (95% CI^**+**^)	***p***-value	Adjusted RR (95% CI^+^)	***p***-value
**Parity**				
Multiparous	Ref		Ref	
Primiparous	1.48 (1.20–1.82)	< 0.001	1.46 (1.18–1.81)	< 0.001
**Gestational age**				
Term	Ref		Ref	
Preterm	0.45 (0.33–0.60)	< 0.001	0.77 (0.51–1.17)	0.221
Post term	1.57 (1.19–2.07)	0.001	1.45 (1.09–1.93)	0.009
**Birthweight (kg)**				
2.5–3.5	Ref		Ref	
< 2.5	0.48 (0.36–0.63)	< 0.001	0.69 (0.48–1.01)	0.051
> 3.5	1.28 (1.03–1.60)	0.027	1.28 (1.02–1.61)	0.030
**Residence**				
Rural	Ref		Ref	
Urban	1.33 (1.09–1.63)	0.004	1.29 (1.05–1.58)	0.015
**Indications for IOL**				
PROM	Ref		Ref	
Postdates	2.96 (1.71–5.12)	0.053	2.00 (0.99–4.04)	0.053
**Methods of Induction**				
Oxytocin	Ref		Ref	
Prostaglandins	0.55 (0.37–0.82)	0.003	0.67 (0.44–1.03)	0.065
**Body mass index**				
Normal	Ref			
Underweight	0.97 (0.77–1.21)	0.105		
Overweight	0.86 (0.68–1.11)	0.246	–	–
Obese	0.81 (0.62–1.05)	0.111		
**Maternal age**				
< 24	Ref			
24–35	0.97 (0.77–1.21)	0.780	–	–
> 35	0.89 (0.65–1.21)	0.450		

*Ref* Reference category, ^*+*^*CI* Confidence Interval

## Discussion

In this study, CS following labour induction was observed in about a quarter of all deliveries and it was associated with primiparity, high birth weight and urban area locality. These results are in line with the finding of studies in Ethiopia and Pakistan [22–24]. Primiparous women had an increased proportion and yet at more risk of CS delivery compared to multiparous. Similar findings have been reported in studies in the United States and Nepal [25, 26]. It is possible that, since primiparous women have no labour experience, the appropriate rate of cervical collagen fibre dissolution is rather hard to attain compared to women with multiple labour experience [27]. We found a high birth weight delivery being at risk of CS delivery. These findings are comparable with those of a study in the Netherlands where babies born with high birth weight had twice the risk of ending up with CS delivery after IOL [28]. Similarly, the study done is Saudi Arabia and Ethiopia reported an increased risk of CS delivery among mothers with big babies compared to those whose babies had normal weight [23, 29]. The increased risk of CS on high birth weight infants may be explained by the high risk of labor obstruction that may be caused by shoulder dystocia which happens when the baby’s anterior shoulder gets caught above the mother’s pubic bone, leading to complications including brachial plexus injury or clavicle fracture, vaginal tears, and excessive bleeding. This obstruction eventually leads to failure in vaginal delivery and hence necessitate emergency CS delivery [28]. In addition, the facility is called to improve strategies for early detection of cephalopelvic disproportion as a good practice in monitoring labor progression using available tools like WHO-labor curve or Friedman’s curve. Deliveries from women who reside in urban areas of northern Tanzania were 29% more likely to experience CS intervention after IOL compared to those living in rural areas. In addition, the current study appreciates more than half (*n* = 259) of study participants who resided in urban, were either obese or overweight. This may be explained by the fact that women residing in urban areas may be more physically inactive than those residing in rural locality hence more likely to be either overweight or obese. As a result, vaginal delivery after IOL may be unsuccessful due to mechanical obstruction of labor caused by accumulation of adipose tissues in woman’s abdomen which in turn leads to fetal distress and eventually imposing a need for CS delivery [30]. More studies are called to analyze relationship existing between mother’s residence and BMI. The effect of IOL indicators were also observed. The study found that, post-date pregnancies (> 42 weeks) had 45% increased risks of experiencing CS after IOL compared to term pregnancies. Though the main cause of postdate pregnancy is still debatable, prior literature shows that postdates pregnancy has been associated with history of postdate pregnancy, maternal obesity, sulfatase deficiency in placenta, advanced maternal age, genetic predisposition, primiparity, central nervous system abnormalities, and fetal anencephaly [31, 32]. Our findings were in line with a study conducted in Sweden found that one of the potential risks of inducing women who are post-dates is an emergency CS where the risk increases [33]. We think that CS following IOL in post-date pregnancies is because post term infant tend to have an increased weight hence making it even harder to attain vaginal delivery [34, 35]. It is also widely believed that there is a decline in placental function after 40 weeks of gestation and the fetus is subjected into an increasingly suboptimal environment, placental insufficiency, meconium aspiration, fetal distress in labor, acidosis, polycythemia, and cephalopelvic disproportion. These factors may call for a need of emergency cesarean delivery [35].

There are limitations in interpreting the findings of this study. While it is known that cervical ripeness has a significant influence on the attainment of vaginal delivery after IOL [36], this study could not assess its role in predicting CS delivery following IOL due to its absence in the institution’s database. We call upon prospectively designed analyses on the role of this attribute in predicting CS delivery in this institution. In addition, the study calls upon improvements in data management and data entry at the institute’s birth registry unit.

## Conclusion

The prevalence of CS among induced deliveries at the facility is high and it is associated with primiparity, high birth weight, post-datism and urban area locality. Assessment of these factors and preparation for alternative delivery mode prior to IOL intervention is warranted to reduce adverse pregnancy outcomes related to emergency CS delivery.

## Data Availability

The data used/or analyzed during the current study is available from the corresponding author on a reasonable request.

## Abbreviations

BMI: Body Mass Index
CI: Confidence Interval
CS: Caesarean Section
IOL: Induction of Labor
KCMC: Kilimanjaro Christian Medical Centre
P: Probability
PROM: Pre-labor Rapture of Membrane
RR: Relative Risk
SD: Standard Deviation
SDG: Sustainable Development Goals
WHO: World Health Organization
